# Thoracic organ machine perfusion: A review of concepts with a focus on reconditioning therapies

**DOI:** 10.3389/frtra.2023.1060992

**Published:** 2023-03-22

**Authors:** Mitchell J. Wagner, Sanaz Hatami, Darren H. Freed

**Affiliations:** ^1^ Department of Surgery, University of Alberta, Edmonton, AB, Canada; ^2^ Department of Medicine, University of Alberta, Edmonton, AB, Canada; ^3^ Department of Physiology, University of Alberta, Edmonton, AB, Canada; ^4^ Department of Biomedical Engineering, University of Alberta, Edmonton, AB, Canada; ^5^ Alberta Transplant Institute, Edmonton, AB, Canada

**Keywords:** machine perfusion (MP), ex-situ organ perfusion, reconditioning therapy, ischemia reperfusion (I/R) injury, oxidative stress, mesenchymal stem cells, hypothermic machine perfusion (HMP), normothermic machine perfusion (NMP)

## Abstract

Thoracic organ transplantation, including lung, heart, and heart-lung transplants are highly regarded as gold standard treatments for patients suffering from heart failure or chronic end stage lung conditions. The relatively high prevalence of conditions necessitating thoracic organ transplants combined with the lack of available organs has resulted in many either dying or becoming too ill to receive a transplant while on the waiting list. There is a dire need to increase both the number of organs available and the utilization of such organs. Improved preservation techniques beyond static storage have shown great potential to lengthen the current period of viability of thoracic organs while outside the body, promising better utilization rates, increased donation distance, and improved matching of donors to recipients. Ex-situ organ perfusion (ESOP) can also make some novel therapeutic strategies viable, and the combination of the ESOP platform with such reconditioning therapies endeavors to better improve functional preservation of organs in addition to making more organs viable for transplantation. Given the abundance of clinical and pre-clinical studies surrounding reconditioning of thoracic organs in combination with ESOP, we summarize in this review important concepts and research regarding thoracic organ machine perfusion in combination with reconditioning therapies.

## 1. A brief history of thoracic organ transplantation and current challenges

It wasn't until the sixties that heart and lung transplantations took a leap forward into the clinical setting, after decades of animal research. In 1963, Hardy and Webb undertook the first lung transplant, whereby a man suffering from left lung bronchial carcinoma received a replacement. In one of the most publicized medical events of the 20th century, Christiaan Barnard performed the world's first human to human heart transplant in 1967 (1–4). One year later in 1968, D.A. Cooley performed the first heart-lung transplant (1, 5, 6). Though the recipients of such transplants quickly experienced complications, these monumental events brought to life the past decades of experimentation and demonstrated the potential of transplantation as a therapeutic option. The immune system presented itself as a barrier to the success of these patients. It wasn't until chemical immunosuppression, as opposed to sublethal irradiation, emerged as an approach that would vastly increase the one-year survival of transplant patients. This technique began with the use of drugs like 6-mercaptopurine and azathioprine plus steroids before cyclosporine was approved for clinical practice in 1984 (1, 4, 7, 8). Currently, the International Thoracic Organ Transplant Registry reports that upwards of 115,000 heart transplants occurred worldwide between 1990 and 2015, and that median survival from most recent data was almost 15 years following transplant (9). Similarly, lung transplants have seen a steady increase from roughly 11,000 transplants done between 1992 and 2000 to roughly 34,000 being done from 2010 to 2018 (10). This data emphasizes how far thoracic transplantation has come in terms of safety, efficacy, and acceptance amongst health centers—in fact, heart or lung transplantation is now considered a “gold standard” of treating late stage heart failure or terminal respiratory disease respectively (5, 11–13). Nonetheless, a current challenge of thoracic organ transplantation (and essentially all other transplantable organs), is reconciling organ demand with the availability of organs (7, 14). This can be done in a variety of ways: by increasing the utilization rate of organs procured from standard sources, liberalizing traditional donation criteria, lengthening the period of viability post procurement, and reconditioning organs that would otherwise be discarded. Machine perfusion is a promising technology which seeks to provide a platform for the dire demand for organs to be better addressed by solving the limitations of current post-procurement organ storage standards. This review endeavors to summarize concepts in machine perfusion for the heart and lung, with a focus on reconditioning therapies delivered *via* the platform.

## 2. High organ demand: factors limiting the supply of quality organs for transplant

One such limiting factor is that donor sources of organs meeting traditional donor criteria are becoming smaller over time as society and medicine evolve. In the early days of transplantation, organs for transplant were procured from donation after circulatory death (DCD) donors, though donation after brain death (DBD) quickly overshadowed DCD donation (4, 13, 15). It has been long appreciated that decreased ischemic times lead to a lower risk of primary graft dysfunction post transplant, being demonstrated in recent clinical studies (16–18). For this reason, as well as management practices which stabilize inflammatory and hormonal processes before procurement, organs from DBD donors are regarded as optimal (19, 20). However, improvements in road and vehicle safety, as well as improved management of conditions that result in brain death continue to decrease availability of donors from this source (11, 20). Furthermore, improvements in medicine have also impacted the DBD pool: the primary cause of brain death has shifted from traumatic brain injury to brain hypoxia. Median age, and incidence of co-morbidities have also increased, signalling a decrease in numbers but also quality of organs from this source (4, 11, 15). The growing push to widen the donor pool as well as a growing sentiment that traditional donation criteria restrict organ supply has prompted a liberalization of traditional donor criteria, introducing the “marginal” or “extended criteria” donor (ECD) (14, 21). The International Society for Heart and Lung Transplant (ISHLT) reported that between 2005 and 2018, an additional 20,000 lung transplants were undertaken due to this widening of traditional criteria (22). A study of marginal heart donation found similarly that marginal donations could yield a 37% increase in transplant volume with similar risk-adjusted mortality to standard donation (23).

Along the same line, DCD donation has been met with renewed interest. Despite the increased ischemic burden due to the mandatory “standoff period”, it has been reported that an additional 149 suitable heart donors could have been utilized over 2011–2013 at an institution in the UK if DCD hearts were considered, an unrealized 30% per year increase in cardiac transplants at that centre (24). Furthermore, the same group showed that DCD hearts were non-inferior to DBD hearts based on 30-day survival (25). Similar findings have been reached with regards to DCD lung transplants. In a study of DCD lung transplants, one year survival for 67 DCD lung transplants was 97% vs. 90% for 503 DBD transplants (26). Even if DCD heart and lung donations may perhaps only yield a short-term solution to patients on the waiting list, they represent a promising extra source of donor organs. Transplant centers within North America have already begun to make use of this extra donor source: within Canada as of 2021, 26% of locally retrieved organs were DCD status (27).

A second source of limitation on organ supply is the succession of pathophysiological insults experienced by such organs which are associated with donor morbidities, as well as procurement and storage of the organs. These insults inevitably limit the period with which the organs can stay viable before they are donated and threaten primary graft dysfunction, which can necessitate a replacement for the newly received graft (28). A short viability time following procurement limits utilization rates, makes highly matched donation difficult, and restricts donation to local donors (11). The experienced onslaught begins before the organ is even procured. Both DBD, and to a greater degree DCD donation, are associated with the induction of a catecholamine storm. This has been associated with acute structural damage to the thoracic donor organs (13, 29, 30), demonstrated by the induction of apoptotic and necrotic damage to the myocardium, and compromise of the endothelium (31, 32). During procurement, all organs undergo a period of global ischemia followed by ischemia-reperfusion injury (IRI) upon donation. The cellular pathophysiology and result of IRI has been well reviewed (13, 28, 33–35). In brief, a lack of oxygen supply to the procured organ necessitates anaerobic metabolism, quickly depleting the cells of ATP needed to maintain ionic homeostasis (13, 28). As primary transport begins to fail, the controlled movement of protons, sodium, and calcium becomes dysregulated, and this eventually results in calcium overload in the cytosol. Subsequently, DNA and protein-damaging reactive oxygen species (ROS) are generated, and calcium dependent signalling is induced, triggering highly inflammatory necrosis and apoptosis (33–37). High osmolarity within the cells can also lead to the formation of edema, which further impairs function (25, 38). Reperfusion, while the only antidote for ischemia mediated pathology, only serves to briefly exacerbate its effects: the washout of formed proton gradients across the cell membrane during ischemia leads to further increased calcium intake. Reintroduction of oxygen to the cells, which have now built-up anaerobic metabolites and depleted their antioxidants, generates more deleterious ROS (13, 33–35).

Should the supply of organs be maintained or even increased, the field will need to devise methods to best accommodate the ever-evolving donor pool and the accompanying pathologies which threaten organ viability. Machine perfusion, which began as research tool for physiology, has been adapted as an answer for the limitations that threaten the supply of donor organs.

## 3. Approaches to thoracic organ preservation

### 3.1. Static cold storage of thoracic organs

The ability to preserve organs for an extended period following procurement without loss of function represents the holy grail of organ preservation and has been the subject of many investigations in recent years. Static cold storage (SCS), a mainstay of organ transplantation, stores organs in a non-functioning manner at 4°C. The provision of hypothermia to the ischemic organ is done to 1) slow rates of metabolism which fuel processes that degrade vital cellular proteins, and 2) to slow the lysis of lysosomes containing autolytic enzymes (35). Though SCS requires minimal effort and is inexpensive, hearts may only be preserved for a maximum of 6 h before the risk of primary graft dysfunction becomes intolerable. This is despite the delivery of a cardioplegic solution that reduces oxygen consumption (11, 15, 39, 40). Similarly, lungs which have been stored in this manner are used given that the preservation period is no more than 8 h, though retrospective study has showed no significant difference in outcomes when preserved by SCS for 12 h (41). The slowed onset of anaerobic metabolism *via* lessened metabolic requirements of the lungs and lingering oxygen within the alveoli can perhaps explain the lengthened period of viability in comparison to the heart during SCS. Given the cold ischaemic stress that thoracic organs are subject to during SCS, the method is not suitable for extended criteria nor DCD donations; their inability to tolerate further damage during cold storage and an inability to evaluate the functionality of the organ during storage present further limitations to this method (13, 37).

While SCS is currently the standard of care, machine perfusion (MP) has been developed to improve on many of its drawbacks, with considerable success. This is demonstrated by the number of clinical protocols which now exist for ex-situ lung perfusion, namely the Toronto, Lund, and Organ Care System (OCS) protocols (42–44). The OCS also currently represents the only clinically available method for ex-situ heart perfusion (45). Preliminary studies by Wicomb et al*.* and Hassanein et al*.* in the 80's and late 90s respectively signaled a growing appreciation that preservation of the donor heart entailed keeping the organ supplied with continuous nutritional and ionic support (46–48). These initial studies, contrasting in their approach, exemplify the two main frontiers of heart machine perfusion: hypothermic machine perfusion (HMP), whereby the heart is kept at low temperatures in a non-working mode (Langendorff perfusion); or normothermic machine perfusion (NMP), whereby perfusion is undertaken at room temperature to 37°C in either non-working or working modes. As with clinical protocols developed for the lungs, there is a variety of experimental and clinical approaches that are accompanied by a plethora of formulations of the perfusion circuit and perfusate composition and come with their own advantages and disadvantages. For ESHP, HMP was first investigated as an upgrade to static cold storage, with NMP being explored later in the history of the field of heart MP. Contrastingly, all ESLP protocols proceed at normothermia, with cryopreservation techniques being regarded as a future direction (37). Though not exhaustive, the benefits and disadvantages of each of these approaches is discussed in more detail in subsequent sections.

### 3.2. Normothermic machine perfusion (NMP) of the thoracic organs

In comparison to HMP, NMP keeps the heart in a semi-physiologic state at ∼37°C. The provision of a working mode has been achieved by our group, enabling functional parameters to be measured in real time by varying pump flow (49). However, clinical ESHP protocol such as the OCS protocol perfuse the heart in a non-working, unloaded state which precludes functional assessment (45). Normothermia is also the premise of the Toronto, Lund, and OCS clinical lung perfusion protocols, which were developed to enable further preservation and functional assessment of donor lungs (44). Hassanein and colleagues were among the first groups to publish on ex-situ heart perfusion at normothermia using a blood-based perfusate, finding that in comparison to SCS, hearts perfused at normothermia benefitted from preserved contractile, metabolic, and vasomotor function, with decreased edema formation over 12 h (48). Likewise, perfusion of the lungs at normothermia with an optimally osmotic perfusate can prevent pulmonary edema, preserve PaO2/FiO2 ratios, and histologic structure in comparison to SCS (42). Theoretically, NMP enables better preservation of donor organs than SCS, or HMP as applicable to ESHP, by subverting the period of cold ischemia which perpetuates time dependent reperfusion injury and resultant metabolic dysfunction at low temperatures. This allows better preservation of organs, especially those from extended criteria or DCD donors (50).

Beyond the ischemic insults that accompany procurement, NMP enables a suite of benefits that may be harder to realize on a HMP platform. NMP is more compatible with blood based perfusates in comparison to HMP. This compatibility is due to the fact that in hypothermic settings, blood must be diluted to prevent coagulopathies resulting from increased viscosity at low temperatures. Furthermore, the dissociation of oxygen from hemoglobin is attenuated at low temperatures, becoming indissociable at 12°C (11, 51). Blood is thought to offer a more physiologic delivery of oxygen *via* red blood cells (RBCs) and has the additional nutritional and oncotic benefits of plasma components in comparison to synthetic solutions used in the setting of HMP (11, 52). The Lund and OCS protocols for ESLP take advantage of this, with RBCs being a component of their perfusate at 10%–25% hematocrit (37). Near physiological metabolism at higher temperatures enables the prospect of dynamic, responsive management *via* pharmacological agents and hormonal support, an especially pertinent benefit for suboptimal donor organs (11, 53). Normothermia, while complimentary to responsive management, also lends itself to reconditioning approaches working as intended, given that gene expression profiles and the functioning of enzymes or proteins can be altered at cold temperatures. Increased temperatures also make it a better platform for delivery of novel gene therapies, whereby efficient viral delivery of the target gene is temperature dependent (54). Discussed later in this review, multiple groups have demonstrated that an ex-situ heart perfusion setup at normothermia is able to facilitate thorough transgene delivery and expression throughout the heart using a luciferase expression system utilizing a cytomegalovirus (CMV) promoter (54, 55). This reconditioning modality has been explored to a greater extent on the ESLP platform, with studies experimenting with IL-10 transgene (56–58).

#### 3.2.1. The clinical evidence for normothermic ex-situ heart perfusion

NMP has been generally regarded as the most optimal temperature for which out of body preservation can be affected, with a suite of theoretical benefits compared to other perfusion temperatures. Practically speaking, the provision of machine perfusion to the clinical sphere has also transformed transplant capabilities, with an abundance of clinical trial results published to demonstrate its practicality in effecting greater organ utilization. The only currently clinically available normothermic ESHP apparatus, the Transmedics Organ Care System™, has gone through an abundance of clinical study: non-randomized PROCEED I and PROTECT I trials were conducted in the USA and Europe respectively comparing NMP using the OCS platform with traditional SCS, after which the PROCEED II prospective, multi-center, randomized trial established non-inferiority of the OCS heart platform with SCS (45, 59). Findings reported from the PROCEED II trial found that in comparison to hearts stored using SCS, there was no significant difference in adverse cardiac related events or 30-day graft survival, though there was a significantly increased out of body preservation time (5.4 h on OCS vs. 3.2 h of SCS) (45, 60). While the designation of study endpoints in this trial precludes insight of the myocardial protection effected by NMP, it at least demonstrates its efficacy as a vehicle to effect greater transport distance of procured hearts while decreasing the period of cold ischemia, therefore benefitting organ utilization. It was originally thought that lactate >5 mmol/l by the end of the run could provide sensitive and specific prediction of outcomes following perfusion (61), however it was pointed out that a low lactate level does not necessarily preclude a high risk heart, as demonstrated by a case study by Stamp and colleagues (62, 63). Experimental study by our own group has suggested a very strong correlation of functional parameters with myocardial performance during NMP, however this would require the OCS to support the excised heart in a loaded, working mode, which is a current limitation (11). Regardless, more optimal predictors for graft performance during clinical machine perfusion are warranted.

The OCS Heart EXPAND trial, which was a single arm study to assess the use of OCS within ECD heart transplantation also demonstrated the ability of the OCS to widen the heart donor pool (64). They reported that an additional 75/93 (81%) ECD hearts were successfully transplanted, with a mean OCS perfusion time of 6.35 h and 30-day and 6-month survival rates at 94.7% and 88% respectively (64). This is very favourable to the prospect of widening the donor pool, given that most ECD hearts would not be able to tolerate six hours of SCS. Further to widening the donor pool, additional study has been conducted for DCD heart transplantation as well: a randomized controlled trial comparing DCD transplantation using OCS preservation with DBD hearts preserved with SCS showed a high utilization rate (89%), with survival rates for DCD transplantees and grafts trending higher than control up to 1 year (65). This echoes earlier studies carried out utilizing normothermic regional perfusion by Messer and colleagues, which showed that DCD heart transplants did not significantly differ in terms of survival at 1 year, length of hospital stay, or adverse events related to the allograft (66). Therefore, there exists strong evidence to suggest that NMP is a powerful technique to not only increase the distance between potential donor-recipient pairs, but also to unlock extra supply of hearts *via* ECD or DCD transplants while retaining comparable patient/graft survival outcomes.

These are not the only benefits to be realized, as the OCS platform has also proven beneficial to complex congenital patients and left ventricular assist device (LVAD) recipients. Patients with multiple VAD implantations or congenital patients with complex cardiac anatomy pose a challenge for surgeons to navigate delicate mediastinal dissection quickly to explant the recipient's original heart, therefore increasing the amount of time the donor organ must wait on ice before implantation. However, with the OCS providing continuous metabolic support, performing surgeons can take their time to perform careful dissection without worrying about deleterious time-related cold ischemia pathology (67–69).

#### 3.2.2. The clinical evidence for normothermic ex-vivo lung perfusion

As was demonstrated with the OCS Heart platform, similar conclusions have been arrived at for NMP of the human lung, with techniques like the Toronto protocol enabling reliable preservation for up to 12 h, over the 8 h maximum afforded by SCS (42). The INSPIRE trial demonstrated similarly that EVLP of the human lungs is non-inferior and even results in a seemingly lower amount of primary graft dysfunction (PGD) grade III after 72 h (70). The NOVEL trial demonstrated that EVLP can enable safe evaluation of donors, enabling 216 donors to be evaluated. Over half of these were transplanted, with non-inferior 1 year survival and indifferent levels of PGD grade III (71). Along with the single arm EXPAND (72) trial, which demonstrated the ability of EVLP to support ECD and DCD donors with a sizeable proportion foregoing PGD grade III, these trials demonstrate EVLP's ability to safely assess questionable allografts before transplantation, which represents a significant advantage over cold-storage for ECD or DCD grafts. Incorporation of EVLP into practice has already significantly impacted the quantity of lung transplants performed at some institutions: for example, Divithotawela et al. reports a high conversion rate of EVLP treated donors, and a steady increase in lung transplants being performed at their program in Toronto despite a steady number of available donors. In their program from 2008 to 2017, 230/936 (24.6%) lung transplants were performed after EVLP with comparable long term outcomes, despite the EVLP group being more fraught with predictors of transplant failure (73). This is reinforced by a meta-analysis of 20 published articles including roughly 2,500 lung transplants done *via* EVLP which concluded that EVLP increased the utilization of marginal donors, reduced total ischemia time, and extended preservation time (74).

#### 3.2.3. The cost effectiveness of NMP and the impact of machine perfusion on the future of institution level transplantation process

Two significant downsides of NMP in comparison to SCS is that NMP is expensive, and technically challenging: per heart transplant, it has been cited that the OCS heart apparatus costs anywhere from $38,000-$55,000 USD for single use components, with a console costing about $275,000 USD (75). Another study reports a staggering cost of around $80,000 USD per use, not including the cost of at least five staff members required to work with the device (68). These numbers are similar in magnitude to estimated costs per transplant utilizing EVLP, which suggest that the net cost (including disposable supplies) would be in the neighborhood of $20,000–60,000 USD (76). While there exists very little study on the cost-effectiveness of ESHP, studies of EVLP have deemed the method cost effective at the institutional level, facilitating faster progression to transplantation without a significant increase in cost (76, 77). It is important to note that these studies focus mainly on device/equipment costs, but do not account for the high labour costs which are necessitated by the technical challenges and expertise needed to effectively run these machines. Demonstrating not only the preservation efficacy, but also the economic viability of machine perfusion, is an important step towards widespread clinical implementation.

The prospect of EVLP, and more widely, machine perfusion, has been proposed to enable a reorganization in the way that transplants are done currently. Akin to the development of centralized blood banks whereby quality control and processing are done before distribution to recipients of transfusions, in the future, organs could be procured and stored at centralized organ repair centers. The expertise, diagnostics, and reconditioning needed to effect reconditioning *via* machine perfusion would be conducted at a centralized location before distributing the donor organs back to hospitals that would transplant the organs into recipients (78). Thus, the significant amount of inefficiency that has developed alongside the field of transplantation would be replaced with a more centralized, orderly, and optimized process for organ transplants (78).

### 3.3. Subnormothermic machine perfusion of the thoracic organs

Subnormothermic machine perfusion (SMP) acts as a middle ground between NMP and HMP, taking place at ∼20–34°C (79). Thus, the method takes advantage of decreased metabolic kinetics to effect tissue preservation, yet in contrast to HMP allows for optimal blood based perfusates since temperature is high enough to avoid impaired oxygen dissociation and potential coagulation (11, 52). Though SMP may superiorly facilitate the use of blood-based perfusates in comparison to HMP, temperatures below that of NMP would allow for greater oxygenation of a crystalloid perfusate, given that gases are more readily dissolved in colder fluid. This is evidenced in one study comparing subnormothermic EVLP with normothermic EVLP, whereby lungs were perfused at 28°C were reported to benefit from superior oxygenation (80). Thus, this may represent an opportunity for acellular crystalloid perfusates to be used to greater effect, subverting the immunogenic, thrombotic, and hemolytic disadvantages of blood based perfusates (52). Furthermore, perfusion at a temperature that is closer to physiology enables more relevant assessments of function, particularly for the liver or kidneys, which compose the majority of studies utilizing SMP (79). Machine perfusion at subnormothermic temperatures is an area of research that is lacking in the context of the thoracic organs, however some studies have found promising results for lungs: Arni et al. reports that in comparison to normothermic EVLP, lungs benefitted from significantly lowered pro-inflammatory cytokines and chemokines (80). Furthermore, the same group reported in a comparison of different perfusion temperatures that 25°C perfusion resulted in decreased histological injury compared to SCS and TNF*α* signalling, though no difference in PaO2/FiO2 ratios was seen between groups (81). These studies are limited in that perfusion was limited to 4 h, therefore subnormothermic EVLP's ability to effect longer term preservation in relation to HMP or NMP approaches is still unknown. Further study, namely during longer time periods up to 12 h, is required to fully elucidate the benefit of SMP in relation to HMP or NMP and identify a potential clinical niche for its application in ex-situ heart or lung perfusion.

### 3.4. Hypothermic machine perfusion of the donor heart

Of the thoracic organs, hypothermic machine perfusion (HMP) has traditionally been used for heart preservation. Generally, HMP keeps the ex-situ heart in a non-beating state, similar to SCS, typically at temperatures between 4 and 8°C. HMP quickly gained appreciation by investigators as an improvement over SCS, given that the heart could be maintained with more physiologic delivery of nutritional requirements and washout of metabolic wastes while still providing myocardial cooling (82). Wicomb et al. demonstrated the potential for HMP to preserve donor hearts in the early 1980s. The group showed that pig hearts retained a level of cardiac output and stroke volume comparable to those of freshly excised hearts, being significantly preserved compared to hearts kept on classic SCS after 24 h of preservation time (47). It was also noted in a canine model that oxidative stress as measured by 8-oxoG (a marker of ROS mediated DNA damage) was lessened during HMP (83). Further investigation showed that in comparison to SCS, HMP was shown to better preserve endothelial structure as marked by reduced levels of endothelin-1 (ET-1, a marker of endothelial damage), as well as a lack of microscopic ultrastructural damage observed after 4 h of perfusion (82, 84). Better ultrastructural characteristics were also shown in a comparison of hypothermic perfusion methods (85). Perfusion of porcine hearts for 4 h resulted in lower lactate levels, reduced AMP/ATP ratios, and higher phosphocreatine/creatine ratio in addition to preserved functional parameters, showing that HMP provides superior metabolic outcomes to SCS (86).

Despite a plethora of evidence of superiority over SCS, the uptake of HMP into clinical use was limited for a few reasons. Formation of edema due to perfusion is a concern of this technique, given the fact that many studies have reported this (47, 83, 85, 87). The formation of edema can compromise myocardial perfusion by increasing coronary vascular resistance, exposing cardiomyocytes to varying levels of oxygenation (83). Edema has also been associated with worsened diastolic function (12). The episode of cold ischemia imposed by HMP may be unacceptable for some extended criteria or DCD donor organs, which are increasingly being utilized to meet organ demand (12, 13). Another concern is that HMP, as with NMP, is a much more expensive and technically challenging method with regard to the mechanics of the circuit and perfusate. This was exemplified in some studies of HMP. Fitton et al. reported that only 60% of hearts being perfused by HMP were able to be successfully weaned from bypass (83). In a study by Wicomb et al., 6/10 hearts utilizing modified Krebs solution were functionally nonviable due to a complication with the perfusate, whereby the cause would be almost impossible to verify concretely due to the multiplicity of ingredients in the perfusate (46). Thus, the risk of edema and the introduction of a host of variables through HMP represented a great risk to reliability not mirrored by SCS, perhaps adding resistance to clinical uptake. A final disadvantage of HMP is a lack of continuous functional evaluation. Though some apparatuses may allow hearts to be transitioned into working mode for functional evaluation, most rely on periodic assessment by an intraventricular catheter, preventing real-time evaluation of the myocardial function. This is important given the fragile nature of extended criteria and DCD organs being increasingly utilized to widen the donor pool, which require close monitoring and management.

### 3.5. Combining various machine perfusion temperatures: cyclical preservation strategies

Whether machine perfusion is conducted at normothermia, sub-normothermia, or hypothermia, these temperature settings need not be mutually exclusive. A study by Aadil et al*.* utilized a cyclic strategy whereby three periods of 10°C storage were interspersed with periods of 4 h normothermic EVLP at 37°C (88). This method was inspired based off of a prior two studies, one study which showed no significant affect of the lungs was had by a second period of cold storage following EVLP, and a second whereby storage at 10°C effected greater mitochondrial protection perhaps *via* the conservation of protective metabolites (89, 90). They show that this cyclic strategy allows for a preservation of metabolic precursors within the lung tissue, and a decreased expression of pro-cell death gene expression, which potentially contributed to preserved PaO2/FiO2 over the course of preservation (88). The success of this preservation strategy seems to lie in the “recharge” mechanism, whereby processes that would disrupt mitochondrial integrity during periods of cold storage are broken up by periods of EVLP, during which metabolism is able to proceed. This may replace metabolic precursors and protective metabolites that are depleted during cold storage, which in turn averts cellular stress, leading to reduced cellular inflammation and pro-apoptotic signalling (88, 90). Further study is required to characterize this mechanism in more detail; however, the approach unveils an imaginative strategy to combine the benefits of different perfusion temperatures while co-opting protective metabolic processes to effect lengthy preservation. This approach could additionally be translated and optimized for machine perfusion of other organs, and different variations of temperature cycles could be studied to further optimize the method.

## 4. A new window for intervention: reconditioning therapies for the heart and lung during ex-situ preservation

**Figure 1:**
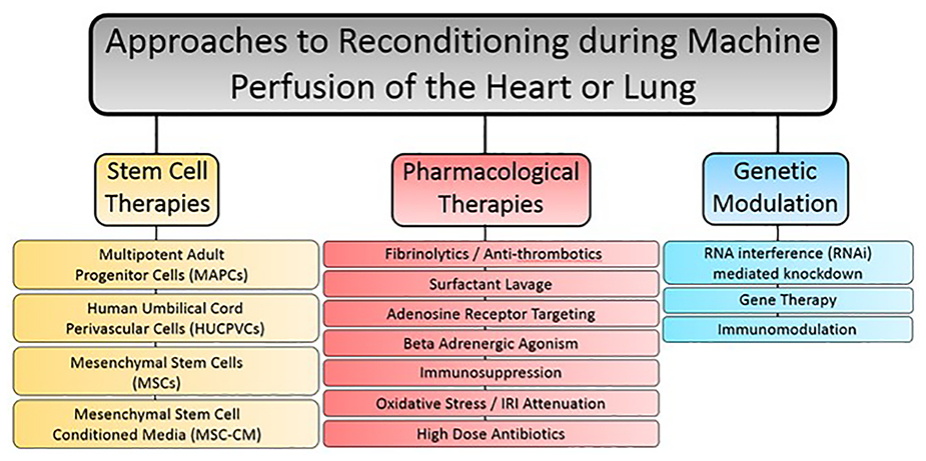
Summary of reconstitution therapy approaches used in combination with machine perfusion for either the heart or lung.

Ex-situ perfusion of the donor heart or lung represents a period whereby the lone organ is separated from the rest of the body tissues, allowing for function improving therapies to be administered in isolation. Given that extended criteria and DCD organs are being utilized to a greater extent in an effort to widen the donor pool, reconditioning therapies will be integral to improving outcomes in patients who receive these grafts (91, 92). Furthermore, though perfusion protocols have proven suitable for ECD and DCD donors, they will not be adequate to fully utilize the totality of available organs for transplant. Reconditioning therapy encompasses a wide variety of approaches to preserve or reinstate the quality of donor organs once procured from the donor. For example, even the provision of a machine perfusion apparatus to continuously provide metabolic support could be considered a reconditioning therapy itself. However, for the purposes of this review, we will consider studies that utilize machine perfusion as a platform for the addition of a medicinal substrate to provide a benefit to the perfused organ (summarized in Figure 1). Typically, reconditioning therapies within this working definition fall under one of three approaches, though the lines may be blurry depending on the study: (1) pharmacological interventions, (2) mesenchymal stem cell-based therapy, and (3) genetic and immunomodulatory manipulations. More recent studies have also focused on the potential of xenogenic cross-circulation to better preserve or even recondition donor thoracic organs, with promising results.

### 4.1. Pharmacological therapies for machine perfusion of donor heart or lungs

**Figure 2:**
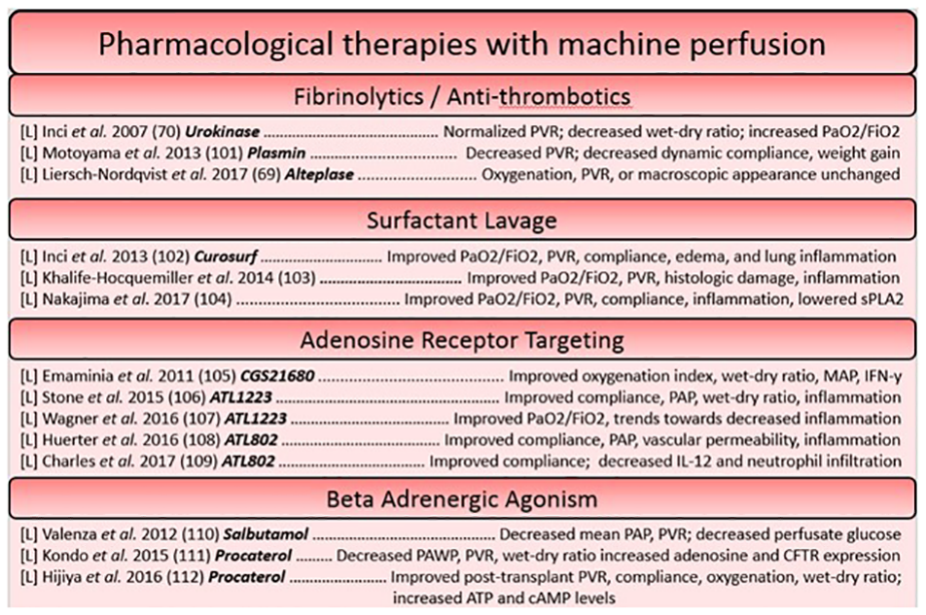
Summary of studies utilizing pharmacological based reconstitution therapies for the heart and lung organized based on approach. Each reference is listed with information regarding the organ studied ([L] = lungs, [H] = heart), the specific agent in bold, and a summary of the key findings.

**Figure 3:**
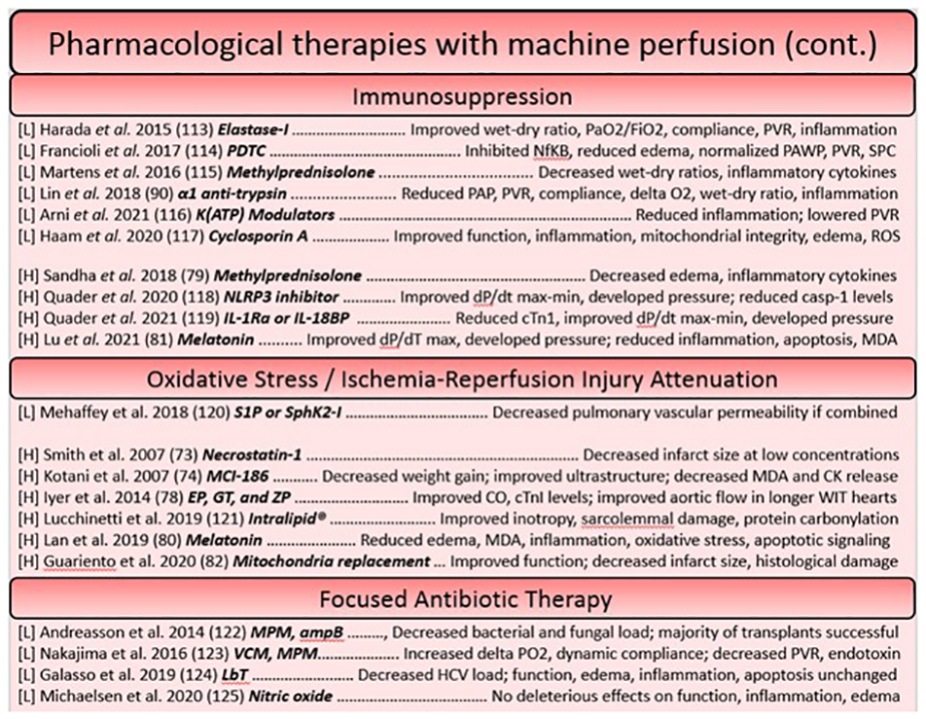
Continued summary of studies utilizing pharmacological based reconstitution therapies for the heart and lung organized based on approach. Each reference is listed with information regarding the organ studied ([L] = lungs, [H] = heart), the specific agent in bold, and a summary of the key findings.

Pharmacological therapies can target a myriad of deleterious processes or pathophysiologies relating to the transplantation process. As long as there exists a validated compound for use, machine perfusion can act as a platform for its delivery to the organ in isolation. A variety of pharmacological reconditioning therapies have been used for the lungs during machine perfusion, including fibrinolytic treatments, high dose antibiotic therapies, adenosine receptor agonists and antagonists, and B-adrenergic agonists, etc. (93). Fibrinolytic treatments work to recondition the lungs on the premise that cardiac arrest produces clots which find their way to the microvasculature of the organ, preventing adequate perfusion (94, 95). One such study hypothesized that the addition of urokinase, an activator of clot-dissolving plasmin, would lead to improved graft function. Indeed, this worked as intended, as function was improved and histologic samples of the lung parenchyma post-perfusion showed a lack of erythrocyte accumulation compared to untreated controls (95). Antibiotic therapies have also been investigated on the ESLP platform, as they are enhanced by machine perfusion. This is due to high doses of broad-spectrum antibiotics not being tolerated by the rest of the body. During isolated machine perfusion of the lungs, high dose antibiotics can be administered in isolation to lungs with a high bacterial load, preventing complications that would arise in off-target tissues (37). Another factor which has been targeted pharmacologically are adenosine receptors. Adenosine, detected by the A2A and A2B G-coupled receptors that are present on a multitude of cells within the lung, is known to mediate polymorphonuclear cell activation and trafficking to the lung during states of ischemia-reperfusion injury (93, 96). By modulating signaling through these receptors using agonists and antagonists to versions of the adenosine receptor, damaging inflammation can be attenuated. Another type of compound studied are B-adrenergic agonists, which act to reduce pulmonary edema. The mechanism of action depends on the agonist used; salbutamol, for example, is thought to reduce glucose within the perfusate which has been correlated to levels of pulmonary edema (93, 97) (Figures 2, 3).

Pharmacological interventions for reconditioning the heart using a machine perfusion apparatus have largely focused on the attenuation of reactive oxygen species generation and targeting processes involved in concerted cell death (98–100), however more modern approaches have sought to dampen the immune response as has been a focus of therapies on the ESLP platform. The idea of pre-conditioning donor hearts has been well explored, as pre-conditioning agents can easily be administered before ischemia through the cardioplegia solution. The lungs on the other hand, necessitate no such vehicle, making pre-conditioning studies rare. This constitutes a potential area of research that could be more widely explored in future in the field of lung preservation. One such study in ESHP used erythropoietin, glyceryl trinitrate, and zoniporide supplements (newly known to activate ischemia postconditioning pathways in a model of large animal DCD donors) in the cardioplegia, finding that the tolerable warm ischemic time could be increased (101). Likewise, another cardioprotective strategy utilized an adenosine-lidocaine cardioplegia, finding that this strategy yielded a significantly increased proportion of stable post-transplantees after NMP in a swine model (53). Other approaches to post-conditioning have included steroids (methylprednisolone) in the perfusate (102), melatonin (an inhibitor of oxidative stress and inflammatory processes) (103, 104), and novel approaches, such as mitochondrial transplant (105). Given that they have found a decrease in caspase-3-like activity, replacement of mitochondria which have lost their integrity during ischemia-reperfusion perhaps prevents the induction of intrinsic signaling for apoptosis and necrosis, ameliorating the detrimental cell loss that threatens functional decline (100). Interestingly, the introduction of autologous mitochondria throughout the DCD heart seemed to not elicit an inflammatory response, and significantly reduced the size of infarcted tissue (105) (Figures 2, 3).

### 4.2. Mesenchymal stem cell therapies for machine perfusion of the donor heart or lungs

**Figure 4:**
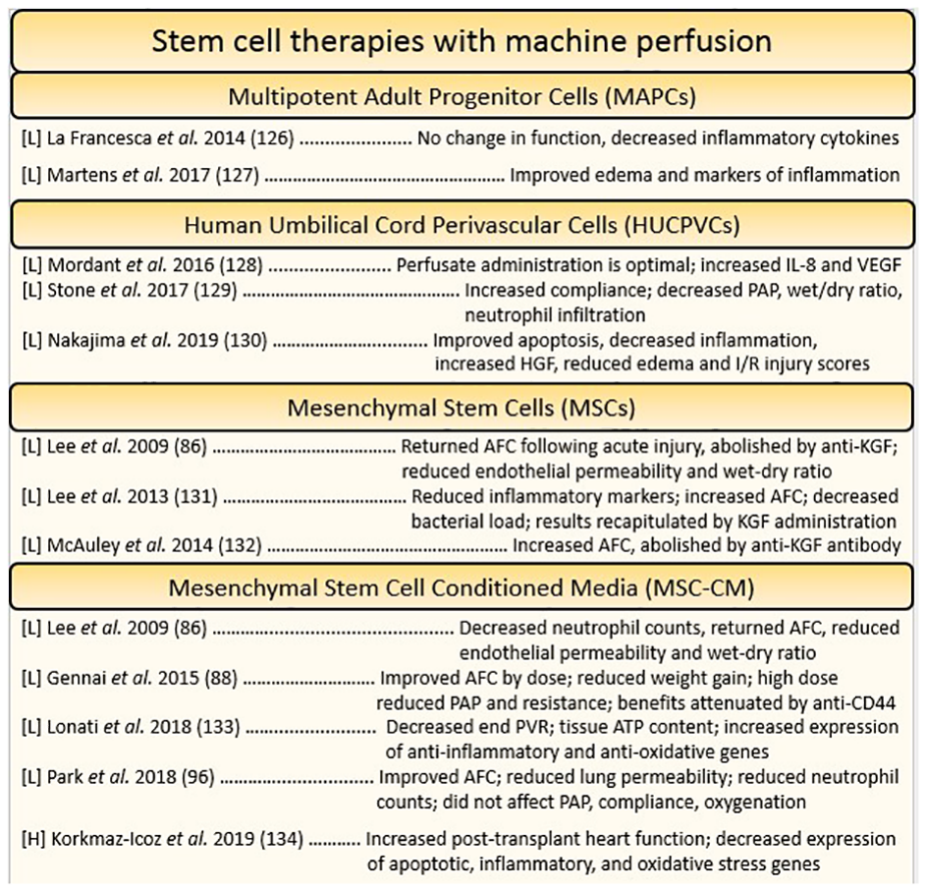
Summary of studies utilizing stem cell-based reconstitution therapies for the heart and lung organized by cell type. Each reference is listed with information regarding the organ studied ([L] = lungs, [H] = heart), and a summary of the key findings.

Mesenchymal stem cells (MSCs) are a type of pluripotent cell which can be found in the bone marrow, adipose tissue, or muscle. It can also be derived from the connective tissue of organs like the liver or heart. They need not be associated with the physical microenvironment of the bone marrow and thus can be cultured ex-vivo with the right supplements (106, 107). MSCs have gained the attention of the machine perfusion community as therapeutic candidates due to their demonstrated immunomodulatory and regenerative effects, and their ability to combat IRI in transplanted organs (91, 92, 108). It was initially thought that stem cell administration yielded engraftment and subsequent differentiation into new cells, however MSCs have been found to primarily elicit their effects in paracrine fashion (91, 109). Secreted extracellular and micro-vesicles (EVs and MVs respectively) contain a suite of growth factors, signaling proteins, and even genetic material (miRNA) which have profound effects on nearby cells (91, 109–111). Investigations of the immunomodulatory effects of MSCs have demonstrated their ability to reduce expression of inflammatory chemokines and cytokines perhaps *via* the suppression of NfKB. Furthermore, MSCs have pleiotropic effects on immune cells, acting to coerce anti-inflammatory activities in macrophages, dendritic cells, and regulatory T-cells (T-regs) while also inhibiting inflammatory signaling through toll-like receptors (TLRs) (91). Micro-RNA (miRNA) from MSC EVs have also been shown to prevent mitochondrial fission, preventing caspase mediated apoptosis and metabolic dysregulation perhaps by inciting anti-oxidant defense (112, 113). Finally, the secretions of growth factors and survival signals further dampen apoptosis and induce regenerating angiogenesis, crucial for the repair of tissue damaged by IRI (92) (Figure 4).

Some caveats to the increasing evidence of MSCs' benefits to machine perfused organs is that administered MSCs suffer from a limited lifespan. It has been found that 10% of administered MSCs could be detected after 6 h in an organ-less perfusion setup (114). With an organ present, blockade in the microvasculature of the organ to be donated can occur due to the innate coagulant properties of MSCs (115). A larger dose, which would be needed to overcome the short lifespan of the MSCs for longer perfusions or to provide greater ameliorative effects, can exacerbate this blockage effect (116, 117). Stem cells are also subject to many variables of which the resultant heterogeneity can pose issues for their reliability as a therapeutic; for example, an MSC population can differ depending on donor sources, the tissues they are harvested from, culturing methods, imposed storage periods, perfusate composition, etc. (118). This currently poses a burden for the generalizability of research results utilizing stem cells and would need optimization should it be translated to clinical transplantation. Another concern raised by investigators of stem cells as a reconditioning therapy during lung perfusion is that the introduction of stem cells can result in tumor formation (119). This has prompted some investigations into the use of MSC-MVs, which may perhaps provide the known paracrine benefits of stem cells without the risk of tumor formation in the donor organ (109, 119). Studies should continue to define the mechanisms through which stem cells benefit perfused organs, such that the secretome of MSCs can be delivered to the perfusate rather than the cells themselves. This would eliminate the issues of lifespan and the potential for embolism within the microvasculature. For a more detailed discussion of the research and applicability of MSCs for machine perfusion of other organs, the reader is directed to the recent reviews of Li et al. and Bogensperger et al*.* (91, 92).

### 4.3. Gene therapy, gene modulation, and immunomodulatory techniques

**Figure 5:**
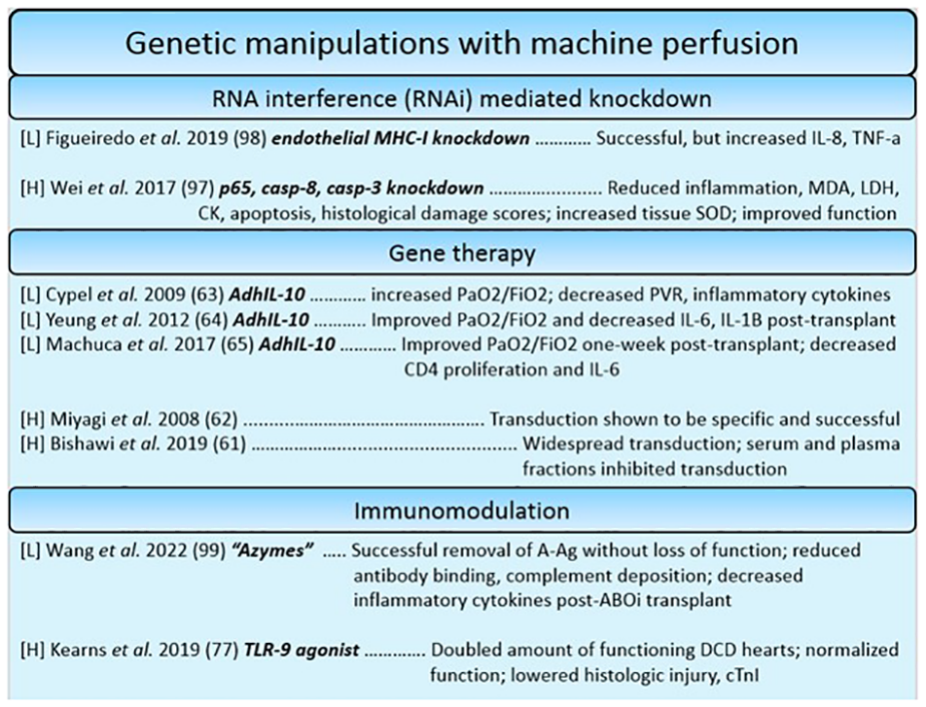
Summary of studies utilizing genetic manipulation-based reconstitution therapies for the heart and lung. Each reference is listed with information regarding the organ studied ([L] = lungs, [H] = heart), the specific manipulations in bold, and a summary of the key findings.

In contrast to the pleiotropic effects that the administration of stem cells has on organs during machine perfusion, gene therapies and gene modulation strategies offer the potential to target a gene or pathophysiologic process with a high degree of specificity. Gene modulation techniques can offer a high range of flexibility, given that specific treatments can be designed to either introduce or overexpress beneficial genes, or silence genes that perpetuate pathophysiology related to transplantation. Most studies using this approach utilize genetic manipulations as a tool to modulate the immune system. One such example utilized CpG ODN 2395, an oligomer of phosphorothioate-linked cytosine and guanine nucleotides. These oligomers work to ameliorate myocardial inflammation by acting as agonistic ligands for toll-like receptor 9 (TLR-9), immune receptors responsible for detecting damage associated molecular patterns (DAMPs) which are released during ischemia related cell damage (120, 121). By pre-treating donors with CpG ODN, cross-tolerance of resident immune cells to agonistic DAMPs is induced, attenuating myocardial inflammation during ischemia. Indeed, they found that pre-treatment was beneficial, almost doubling the amount of physiologically acceptable DCD hearts in their model (121). However, this strategy of pre-treating however may be limited by the ethics of organ donation. Another example of such immunomodulation is the introduction of IL-10, a beneficial cytokine that has a range of functions from being an anti-fibrotic to immunoregulatory factor, *via* a viral vector to donor lungs. It is thought that increased levels of IL-10 act to prevent deleterious inflammatory and fibrotic injury resulting from ischemia (56–58) (Figure 5).

For ESHP, apoptotic and inflammatory genes were targeted for silencing. A study found that downregulating complement 3, caspase 3 and 8, and inflammatory factor kB-p65 had a beneficial effect on apoptotic, inflammatory, and functional outcomes (122). This demonstrates that though highly specific, gene modulation techniques can be used additively to silence many genes involved in pathophysiologic processes simultaneously. This approach was also used to target the expression of MHC on lung endothelial cells, the premise being that silencing MHC expression on endothelial cells would make the graft “invisible” to the new host's immune system, ideally reducing immunogenicity and allowing recipients to forego a life-long regimen of immunosuppressants (123). This approach is known as “immunocloaking”. Indeed, it has been demonstrated: one group introduced silencing gene products of swine MHC components beta-2 microglobulin (B2m) and swine-leukocyte-antigen DRa, causing significant downregulation within endothelial cells. Despite the successful silencing, inflammatory cytokines seemed to be increased nonetheless, representing some challenges to be worked out as a preliminary approach (123). In a landmark paper recently published by Wang et al*.* a similar effect has been achieved by glycolytic enzymes. The enzymes were used to effectively remove immunogenic ABO blood group antigens to transform A-type lungs into group O lungs. Despite identical lung function post-treatment, inflammatory cytokines TNF-α and IFN-*γ* were reduced, and the lungs displayed decreased amounts of bound anti-A antibody suggesting decreased antibody-mediated rejection. Further study is warranted to deduce whether replacement of the cleaved antigen over time poses an issue for long term transplantation success (124). However, it seems a promising way to re-allocate lungs such that the differential waitlist periods for different ABO types can be normalized.

While gene modulation techniques, which insofar have focused on immunomodulation, are an active area of research and development, many barriers must be overcome before they may be a practical option for reconditioning. While additive flexibility may pose a therapeutic benefit, it can also impose a biomanufacturing burden should the method be adopted as a mainstay treatment modality. Wei et al. points out that while small amounts of gene product are needed for testing in small animals, humans would require substantially larger amounts of gene product for efficacious suppression or transduction. For efficacious gene transduction, studies in large animals have required up to 50 billion particles (54). To get an idea of the cost of such gene therapies, a one-time gene therapy for spinal muscular atrophy in children, not adults, was reported to cost $2.125 million (125). However, as techniques in biomanufacturing improve and the benefits of economies of scale are capitalized on, this immense cost should decrease over time. Current studies have been pre-treating the whole animal before perfusion; should administration occur through the machine perfusion platform, isolation on the apparatus would significantly decrease the effective amount of tissue to be transfected. At the point of this review, data for transgene expression on the machine perfusion platform has only been made available for relatively short periods following transplantation. It has been shown that IL-10 transgene remained expressed up to 7 days following transplantation, though investigation needs to be undertaken to understand the impact of prolonged gene expression on outcomes (58). Since cells have intrinsic defense mechanisms for repudiating foreign genes such as DNAses and DNA methylation, this imposes a barrier to long term gene modulation (55). Should repeat administrations of the gene product need to be performed in the future, induced humoral and cellular immunity to the vehicle will abrogate the efficiency of gene transduction (55). Not to mention, the organ would also no longer be isolated on a machine perfusion apparatus, rendering subsequent attempts to apply the transgene subject to the inefficiency and lack of specificity afforded by intravenous delivery.

## 5. Xenogenic cross-circulation as an emerging preservation and reconditioning approach during ex-situ thoracic organ perfusion

It is well appreciated that donor organs being preserved ex-situ suffer from steady functional decline, contrasted by the observation that donor organs, once attached to the circulatory system of a recipient, last for weeks. The marriage of the perfusatory apparatus and the circulatory system of a live animal during cross circulation has gained much attention for its ability to recondition donor lungs, and has also been investigated in the heart (126–129). In a landmark study by Hozain et al. human lungs unsuitable for transplant were reconditioned over 24 h of xenogenic cross-circulation, with PaO2/FiO2 ratios increasing a mean of 135 mmHg to a mean of roughly 320 mmHg, marking a significant increase in functional recruitment and thus transplantability (128). They report significantly reduced inflammatory cytokines within the BAL, decreased tissue infiltrate of neutrophils, decreased apoptosis score on histology, and a significant reduction in serum P-selectin (a marker for endothelial damage). Similarly, hearts subjected to continuous xenogenic cross-circulation benefitted from increased ex-situ viability time, reported to be up to 3 days, associated with significantly decreased edema formation (127). Despite these fascinating results, either group reports no discrete mechanism for the observed functional improvements.

There are two hypotheses for the mediated functional benefit to the donor organ in this setting: one, cross-circulation of the plasma allows for function-deteriorating molecules to be filtered out as a consequence of the live animal's hepatorenal system; and two, molecules supplied to the plasma by the introduced body system (eg. nutrients, hormones etc.) maintain vasomotor tone and endothelial integrity such that function of the donor organ is better maintained throughout perfusion (126, 127). Given that physiologic maintenance of organ function *in-vivo* is the consequence of homeostatic mechanisms facilitated by organ-organ interactions, it is a small leap to assume that each of these hypotheses likely contributes to the observed benefit during ESOP.

Research is ongoing to decipher the molecules being either subtracted from or added to the perfusate. Hemofiltration shows much promise as an approach to investigate the subtractive hypothesis, as important substrates can be monitored over time within the perfusate and hemofiltrate and associated with functional benefits, without confounding additions by unknown sources, as would be the case with cross-circulation. For ESHP, Johnson et al*.* reports that in comparison to controls, hearts subjected to continuous hemofiltration during ESHP showed decreased edema, reduced histological damage scoring, and a lack of increase in coronary resistance. It may be that hemofiltration removed edema-inducing factors which decreased coronary occlusion, therefore mitigating anaerobic respiration and myocardial damage over the course of the run (130). The prospect of hemofiltration in combatting induced edema has also been studied in the context of the lungs, where hyper concentration of the perfusate by continuous hemofiltration without fluid replacement was able to mediate up to a third reduction in gained lung fluid, though did not affect *P*/F ratios (131). Another hypothesis, alluded to by Nilsson and colleagues, is that hemofiltration may be able to remove free hemoglobin released by hemolysis during perfusion (131). They point out that free hemoglobin is able to scavenge nitric oxide, inhibiting its ability to induce vasodilation; thus, a removal of free hemoglobin by the hemofilter may explain the lack of increase in coronary resistance observed during continuous hemofiltration of donor hearts ex-situ (132, 133). It is also known that free hemoglobin is a cellular toxin, mediating oxidative stress, endothelial cell injury, and inflammation in settings of high hemolysis (134). This sentiment is echoed in cross-circulation of donor hearts, whereby blood based cross-circulation was reported to yield higher injury scores on histology than plasma only cross-circulation despite little other reported differences (126).

Indeed, the washout of cytokines, and perhaps DAMPs, by either hemofiltration or xenogenic cross-circulation also appears to be a valid hypothesis for observed functional benefits. This explanation seems particularly applicable in the study by Hozain et al*.* given that the steady decrease of inflammatory cytokines as measured in broncheoalveolar lavage (BAL) along with reduced leukocyte infiltrate could be most associated with the functional benefit to the lung during cross circulation (128). This is reinforced by other studies, whereby cytokine adsorbers utilized to sequester the inflammatory mileu were associated with improved lung function, edema formation, and reduced incidence of PGD following EVLP (135, 136). It is also known that DAMPs, also released during IRI, steadily increase over the course of EVLP, and are associated with increased TNF-α secretion as a downstream result of DAMP induced signalling (81, 137). It has also shown that M30 and high mobility group box-1 protein (HMGB-1) are significantly increased during EVLP in a group of lungs with PGD grade III (138). Perhaps the deleterious effects of these circulating inflammatory mediators can be averted by their removal during continuous hemofiltration, resulting in better control of inflammatory processes during ex-situ preservation. A more detailed biochemical characterization of the metabolic, inflammatory, and oxidative profile of cross-circulated and hemofiltered thoracic organs is warranted to identify potential mediators of these functional changes. Such identified mediators of functional deterioration during ex-situ perfusion would become obvious targets to effect longer and higher quality preservation of all donor organs, perhaps even beyond the lungs or heart.

## 6. Summary and the future of ex-situ thoracic organ perfusion

As it stands, the field of transplantation is highly opportunistic, catering to the availability of donor organs and the significant restrictions accompanying organ donors that permit transplantations with good outcome. Ex-situ thoracic organ perfusion has been demonstrated to not only constitute a significant advantage over conventional storage methods during transplant, but also holds great potential to revolutionize the way that transplants are approached both institutionally and biomedically. As a way to expand the donor pool, ex-situ organ perfusion setups are increasingly being utilized as therapeutic platforms to increase donor organ function by combatting transplant related pathology, effecting better outcomes and improved preservation. In the future, pathophysiology mediated during the transplant process will be able to be completely reversed by therapeutic options, permitting organs to be not only reconditioned from inviable to transplantable, but even beyond, perhaps acting as a standalone treatment modality. In the perhaps not so distant future, operating surgeons could isolate failing organs and utilize a machine perfusion apparatus as a platform to introduce concentrated therapeutic interventions to the standalone organ before retransplanting. Such therapeutic modalities will be critical in the progression of the field towards the holy grail of indefinite storage. As evidenced by the litany of studies available testing experimental reconditioning approaches, much work has been done; however, more work is needed to understand the biochemical pathology that mediates steady functional decline of organs on the apparatus, and how therapeutic interventions can be applied to combat such pathology. Therefore, the restrictive criteria with which donors must adhere to in order to donate their organs will be further liberalized, as deficits in quality can be compensated through machine perfusion therapy. Combatting the steady functional decline through established therapeutic interventions will allow the field to progress towards the holy grail of organ banking. By way of these investigations, ex-situ organ perfusion symbolizes the prospect of making transplantation entirely elective, a stark contrast to the field of transplantation's current limitations.
